# A Family Systems Perspective on Attachment Security and Dependency to Mother and Father in Preschool: Differential and Reciprocal Effects on Children’s Emotional and Behavioral Problems

**DOI:** 10.3390/brainsci13010035

**Published:** 2022-12-23

**Authors:** Alexandra Iwanski, Lucie Lichtenstein, Fabienne Forster, Céline Stadelmann, Guy Bodenmann, Peter Zimmermann

**Affiliations:** 1 Department of Psychology, Developmental Psychology, Bergische Universität Wuppertal, Gaußstr. 20, 42119 Wuppertal, Germany; 2 Cantonal Psychiatric Clinic St. Gallen, Gynaeco-Psychiatry, Zuercherstrasse 30, 9500 Wil, Switzerland; 3 Department of Psychology, Clinical Psychology for Children/Adolescents and Couples/Families, University of Zurich, Binzmuehlestr. 14, 8050 Zurich, Switzerland

**Keywords:** attachment, dependency, psychopathology, mother, father, family systems perspective

## Abstract

Attachment security and dependency play a decisive role for children’s mental health. From a family systems perspective, reciprocal effects of dyadic attachment to each parent within the same family on child symptomatology may well offer additional insights in developmental processes as parents and children influence each other consistently. This study examined the influence of child-mother as well as child–father attachment security and dependency on maternal, paternal, and observed ratings of children’s emotional and behavioral problems. A total of 124 families with preschool children participated in this study. Attachment security, dependency, and symptomatology of the children were independently observed during home visits. Furthermore, mothers and fathers rated child symptoms. Results revealed promotive effects of attachment security to both parents on observed child symptoms. Furthermore, we found a significant actor effect of child-mother attachment security, as well as a significant partner effect of child–father dependency on maternal ratings of child symptomatology. Attachment security to both parents is promotive for child mental health. The family systems perspective clarifies the meaning of child–father relationships for maternal perception of the own child.

## 1. Introduction

Recent research on child development and adjustment includes fathers not only as informants about their children’s developmental outcomes but also by examining their roles and effects as caregivers [1,2]. Such research often investigates similarities in caregiving characteristics between mothers and fathers in general or the individual effects of each parent’s behavior on children’s developmental outcomes separately [3,4]. However, from a family systems perspective, reciprocal effects of caregiving by mother and father and dyadic attachment to each parent within the same family may offer additional insights into developmental processes as parents and children influence each other’s daily interactions with potential effects on mothers’ and fathers’ caregiving, attachment security or insecurity to mother and father, and other developmental outcomes [5,6,7]. The current study aimed at testing such differential and reciprocal effects of preschool children’s attachment security and dependency to mother and father on observed and reported child emotional and behavioral problems.

### 1.1. Attachment and Psychopathology

#### 1.1.1. Theoretical Assumptions

Attachment theory offers an integrative framework for explaining the role of emotional caregiving experiences for the development of adjustment and psychological wellbeing over the life-span [8]. The relevance of attachment for later adaptation or maladaptation during childhood and beyond is a central topic of attachment research [9,10,11,12,13,14,15].

Attachment theory postulates at least three potential mechanisms linking attachment to later wellbeing or maladjustment. First, attachment as an early stage-salient issue influences the subsequent development of competences (e.g., social competence, behavior regulation) and success in later stage-salient developmental issues. According to this developmental model, success or failure in early stage-salient issues affect the probability of later (mal-)adjustment [9,16]. Second, as shown in the field of developmental psychopathology, secure attachment is a major promotive and protective factor (e.g., through a high self-esteem and internal control convictions) [17,18,19], whereas insecure attachment can be understood as a vulnerability which may increase the probability of maladjustment when children or their families are additionally challenged by risk factors [20,21,22]. Third, early attachment experiences influence the development of internal working models of attachment and the self [8], which control social information processing and emotion regulation and therefore contribute to adjustment or maladjustment [11,23,24,25,26]. These internal working models guide chronic (mal-)adaptive interactions within the close family relationships but also in other social or emotional contexts [25,27,28,29,30]. Thus, attachment theory offers an integrative theoretical framework to understand the effects of early relationships on later adaptation and mental health. These theoretical assumptions have been examined empirically for attachment security to mother and father in early childhood and in preschool either using the Strange Situation Paradigm in the lab [31] or using Attachment Q-Sort (AQS) ratings by parents or observers based on home visits differentiating attachment security (and dependency). The empirical results for both attachment assessment approaches are comparable. 

#### 1.1.2. Empirical Evidence

Theoretically, attachment theory suggests that insecure attachment increases the probability of child’s symptomatology, especially when confronted with critical life events. Indeed, meta-analyses show a robust association between attachment insecurity and children’s externalizing symptoms, d = 0.31; 95% CI: 0.23, 0.40; k = 69; N = 5947 [13], d = 0.49; 95% CI 0.42, 0.56; k = 116; N = 24689 [32], as well as internalizing symptoms, d = 0.15; 95% CI: 0.06, 0.25; k = 42; N = 4614 [33], d = 0.58 (95% CI 0.52, 0.64; k = 165; N = 48,224 [32] (see also [34,35]). Studies using the AQS found the largest effect sizes for the association between attachment insecurity and children’s emotional and behavioral problems, especially for externalizing symptoms, k = 7, *n* = 464, d = 0.70**, CI 0.51, 0.90 [13]. We assume that the AQS as a dimensional attachment measure can capture more subtle variability and fine-grained differences in attachment security compared to an attachment classification approach [36] and can capture both secure base and safe haven behavior in children. Consequently, it is a promising instrument to assess different nuances in attachment security (and dependency) to mother and father.

##### Effects of Attachment to Mother and Father on Child Psychopathology

It is important to note, though, that the vast majority of studies on the association between attachment and child symptomatology focus on attachment to mothers and that studies including fathers or both parents are rare. Thus, the understanding of the relevance of child–father attachment for child adjustment and mental health is limited.

For example, in a recent meta-analysis on the association between attachment insecurity and internalizing symptoms, only three out of 42 studies (7%) assessed attachment to fathers and reported no overall significant effect of attachment insecurity to the father on internalizing symptoms, d = 0.01; 95% CI −0.28, 0.30; k = 3; N = 191 [33]. Similarly, in a meta-analysis on attachment and externalizing symptoms, only one study (1%) considered attachment to father [13]. Therefore, it is important to extend the current empirical literature on the effects of attachment security to fathers on children’s development of emotional and behavioral problems. A recent meta-analysis that specifically focused exclusively on the association between attachment insecurity to fathers and children’s emotional and behavioral problems, based on a still small number of studies, reported relevant mean effect sizes for the association between attachment insecurity to the father and externalizing symptoms, d = 0.37, 95% CI 0.20, 0.55, k = 15; N = 1304, as well as internalizing symptoms, d = 0.17; 95% CI: 0.03, 0.31; k = 12; N = 1073 [37]. Thus, effect sizes of the associations between attachment security to mothers and fathers and child psychopathology are somehow comparable and both attachment relationships play an important role for child mental health, when studied separately and not within a family systems perspective including both attachment relationships. Moreover, as only few studies assessed attachment to father, it is not yet clear whether the AQS attachment security will show similarly higher effect sizes compared to other early attachment measures as has been reported for AQS attachment security to mother [33]. More research is needed.

Besides associations of AQS attachment security and child psychopathology, relations between AQS dependency and child maladaptation are also of interest. The AQS assesses attachment security, as a dyadic relationship construct, as well as dependency, as a more individual charactristic of the child [38]. Rather few studies examine the association of dependency and child symptomatology or maladaptation. In one study, dependency to mother (not to father) was associated with more preschool boys’ classroom anxiety and less competence assessed in teacher ratings. Boys’ dependency to father was associated with less social competence [39]. Furthermore, dependency to mother was associated with shorter sleep duration, interpreted as a sign of sleep problems in infants [40,41]. Thus, attachment security as well as dependency are related to child (mal-) adaptation. This is of special interest as increased dependency could also be a characteristic of children with insecure-ambivalent attachment [42] which has recently been shown to to be associated with increased externalizing symptoms [43].

##### Cumulative Effects of Attachment to Mother and Father on Child Psychopathology

Attachment theory suggests that secure attachment to both parents increases the probability of positive developmental outcomes [8,9,44]. The current debate in attachment research whether the number of a child’s secure attachment relationships affects child development [45] has a long tradition [46]. However, not many researchers investigated additive or compensatory effects of the number of secure attachment relationships on subsequent adjustment [45] differentiating the possible combinations of attachment security and insecurity and compare compensatory and cumulative effects of attachment security to mothers and fathers. Bretherton [47] suggested an “averaging” effect of one secure and one insecure attachment to parents leading to less competence in child outcomes compared to children with two secure attachment relationships. However, children with at least one secure attachment may still show better adjustment in many domains compared to children with two insecure attachment relationships. Verschueren and Marcoen [48] reported in their study with kindergartners that children with secure attachment representations of both mothers and fathers showed fewer internalizing symptoms compared to children with two insecure attachment representations. Children with only one secure attachment representation were in between the two other groups. These results suggest that one secure attachment pattern can compensate for or buffer against the effect of one insecure attachment relationship. A study by Kochanska and Kim [49] also supports the compensation or buffering hypothesis, showing that children insecurely attached to both parents had more behavioral problems (in self-reports and teacher reports) than children with at least one secure attachment. However, attachment security to both parents did not result in less behavior problems. Thus, the results did not support a linear cumulative effect of the number of secure attachment relationships on adjustment. Using the AQS, Boldt et al. [50] also reported a buffering effect of secure attachment relationship to one parent at age two on later behavior problems at age eight in case of low attachment security to the other parent. Recent meta-analytic evidence suggests that only children with two secure attachments in early childhood had fewer internalizing problems compared to children with one or two insecure attachment relationships [51]. The effect was less clear for externalizing problems. However, depending on the age of the assessed outcome variable effects may differ even in the same longitudinal study. In the Regensburg longitudinal study, infant attachment insecurity to mother but not to father predicted peer aggression in preschool [52], but in adolescence, infant attachment to father but not to mother predicted disruptive peer behavior [27]. Thus, studies show effects of attachment to mother or father on later child development. Empirical evidence is not always in favor of cumulative or buffering effects. However, most theoretical models and assumptions and most of the empirical studies on the association between attachment to mother and father and the developmental outcomes primarily consider or examine a dyadic perspective of each individual caregiver–child relationship and its specific effects but do not apply a family systems perspective, assuming that the attachment relationship to one parent may influence the interaction or perception of child’s behavior of the other parent.

#### 1.1.3. Family Systems Perspective on Attachment and Psychopathology

**Figure 1:**
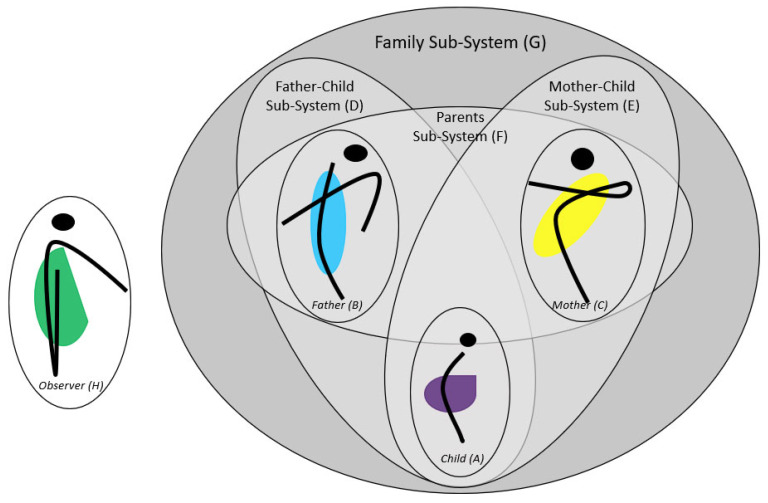
Family Systems Perspective on Attachment and Psychopathology.

Family systems theory understands families as an organized whole with reciprocally interdependent elements [53,54,55,56,57]. Figure 1 illustrates the family systems perspective on attachment and psychopathology.

Each individual (e.g., child (sub-system A), father (sub-system B), and mother (sub-system C)) within a family build its own individual sub-system. Attachment relationships are dyadic characteristics of each dyad within a family system (e.g., father–child dyad (sub-system D), mother–child dyad (sub-system E), and mother–father dyad (sub-system F)). All individuals and dyadic sub-systems are part of the whole family sub-system (G).

This perspective considers that each family member affects the other family members’ feelings, expectations, interpretations, mind-sets, and actual behavior during daily interactions and is affected by them (circular interdependence). As a consequence, individual characteristics (e.g., personality, psychopathological symptoms, attachment representations, see sub-systems A, B, C) as well as dyadic relationship characteristics (e.g., attachment security to mother/father, parental sensitivity, conflicts, see sub-systems D, E, F) are moderated by each family member’s behavior, emotions or evaluations (see Figure 1). Moreover, not only individual or relationship characteristics but also the interpretation by each family member may be affected. Thus, individual behavior, perception, and also psychopathology or mental health can be better understood within the family context [56,58].

There is some empirical evidence for such reciprocal processes in families [59]. Conflicts between parents (parents sub-system, F) may influence parental emotional availability differentially for mothers and fathers leading to differences in children’s attachment experiences with mother (mother–child dyad, D) and father (father–child dyad, E). Thus, the same behavioral variable has different relationship outcomes for the same child (individual sub-system, A) depending on the interaction partner (mother or father). Premarital conflicts between parents longitudinally predicted lower AQS attachment security to mother but not to father, whereas it was associated with higher AQS dependency to father but not to mother. In addition, parental relationship satisfaction was only associated with AQS attachment security to mother but not to father [60]. A study applying an actor-partner-interdependence model (APIM) showed that mothers high in destructive conflict behaviors between parents perceived themselves as less punitive towards their children, whereas this was not the case for fathers. In contrast, paternal stone-walling was associated with less maternal supportive caregiving reported by mothers [61]. Thus, an individual characteristic of the father (individual sub-system B) influences the dyadic sub-system of mother and child (dyadic sub-system D) in the perception of the mother (individual sub-system C) (see Figure 1).

In addition, attachment security to one parent can be associated with only one parent’s appraisal of child problem behavior. In a study with preschool children, only mothers’ CBCL ratings of their children’s mental health problems were associated with child-mother attachment security, but not ratings by fathers or teachers [62]. Interestingly, in the meta-analysis children’s externalizing and internalizing problems were only rated by fathers in 33% of the studies included, and in 20% (externalzing) and 25% (internalzing) of the studies a combined score of maternal and paternal ratings was used [37]. Thus, father’s perspective on child outcomes was seldomly considered, parent ratings are often combined, and the discrepancies in the perception of the child are mainly ignored. However, discrepancies between different informants (e.g., mother, father, teacher) on child psychopathology can be tremendous and informative. Studies including the perspective of both parents are rather rare.

#### 1.1.4. Rater Effects on Child Psychopathology

Research on preschool child psychopathology and clinical diagnosis mainly depends on mothers as informants either using rating scales or clinical interviews. When other informants such as fathers or preschool teachers are included, there is only modest agreement among them [63]. Meta-analytic evidence shows a mean rater agreement for child psychopathology in childhood and adolescence of r = 0.28 [64] with a greater concordance between parents compared to teachers. Studies on preschool children report rater correspondence between mothers and fathers of r = 0.38 and r = 0.35 for internalizing and r = 0.44 and r = 0.49 for externalizing behavior at age three and age five, respectively [65]. However, agreement between parents seems clearly higher than correspondence with external ratings by teachers or examiners. In some studies, father’s rating of children’s internalizing behaviors is the best longitudinal predictor of later child mental health problems [65]. We conclude that including fathers as informants may enhance the validity of child mental health assessments.

Martel et al. [63] suggest in their review that discrepancies between raters may depend on cross-situational variability in children’s behavior depending on situational characteristics, different standards and attributions for appropriate behavior, individual characteristics of each parent and child, observability of problem behavior, and social desirability. As a consequence, parental ratings of child mental health problems may be confounded by parent specific expectations, standards, but also by different interaction styles of mothers and fathers, children’s differential reactivity to each parent, and different interaction contexts. Applying the family systems perspective as mentioned above, each parent’s rating of child behavior also may well depend on the observed interaction characteristics with the other parent. Therefore, it seems necessary to observe children’s problem behaviors in interaction with each parent in comparable situations to disentangle each parent’s subjective evaluation of child problem behavior and the more objectively observed problem behavior.

Studies using observation of child problem behavior in emotionally arousing contexts for the assessment of children’s mental health problems try to introduce more objective criteria in preschool mental health assessment. Studies show moderate associations with maternal ratings of child problem behavior [66,67] and suggest that rater discrepancies between preschool teachers and maternal ratings may partly be explained by the different contexts where they observe the child [68]. Thus, although children’s disruptive behavior with mother and an unknown adult correlated rather high, maternal ratings of interaction with her or an unknown adult showed specific correspondence with each interaction partner in observed interaction [69]. Surprisingly, there are only few published studies on observational assessment of child psychopathology in interaction with the father [70]. Thus, we do not exactly know whether differences in ratings between mothers and fathers can be attributed to differences in interaction styles or contexts between parents or depend on differences in expectations, attributions or standards (i.e., the mind-sets) constructed regarding the child.

According to family systems perspective, more precisely constructivism, mothers and fathers have an individual socially constructed reality of their child’s symptomatology [71]. The assessment of only maternal ratings of child emotional or behavioral problems covers only one subjective construction of symptoms (see Figure 1, individual perspective by the mother (C)). Thus, different constructions were included in the current study by the use of maternal (individual sub-system C), paternal (individual sub-system B), and objective observed ratings of child symptomatology by an external observer (individual sub-system H), who is not part of the family sub-system (G).

### 1.2. Current Study

Prior studies examining the role of attachment relationships as potential promotive factors for child mental health seldomly used a family systems perspective to understand differential and reciprocal effects within families. Studies including both fathers’ and mothers’ ratings and observer ratings of symptomatology are rare. Thus, we wanted to fill this lacuna and used a family systems approach including attachment security and dependency to both parents and three sources of child emotional and behavioral problem ratings. Specifically, we had two main aims in our study:

(1) First, we wanted to examine whether child-mother and child–father attachment security and dependency and reported as well as observed child emotional and behavioral problems are associated. We expected attachment security to mother and father to be significantly negatively and dependency to mother and father to be significantly positively related to child symptomatology [13,32,33], especially to maternal reports as shown in previous research. In addition, we expected objective observation of child symptoms to be notably associated with attachment security to both parents.

(2) Second, we examined potential actor and partner effects of attachment security and dependency to mother and father on maternal and paternal ratings of symptomatology. There is a lack of studies applying an actor–partner-interdependence model in attachment research. We expected significant actor effects of child–mother attachment on maternal ratings as well as actor effects of child–father attachment on paternal ratings of symptomatology, as the relationship quality of one parent–child dyad might influence the other parent’s perception of the child. Moreover, we expected objective observation of child symptoms to be associated with attachment security to both parents even after controlling for parental perceived child symptom reports.

## 2. Materials and Methods

### 2.1. Participants

Data from the “Parental Relationship and Child Development” study, a longitudinal study funded by the Swiss National Science Foundation were used [72]. The study had eleven waves of data assessment. Current analyses are based on data from wave 10 (three years after birth). Couples were accompanied in the transition to parenthood and families were followed until four years after birth, beginning with data collection during the third trimester of the pregnancy of their first child. Participants had to be in a committed mixed-sex relationship for at least one year, had to speak and understand German, and should currently not be in treatment for physiological or psychological disorders or relationship problems. The Ethics Committee of the Philosophical Faculty of the University of Zurich approved the study.

A total of 141 families of the ongoing longitudinal study participated in the observation assessment at wave 10. In one family only father and child participated, in 14 families only mother and child participated, and in two cases video tapes for observations were damaged. Thus, current analyses are based on 124 complete family data sets.

Mothers’ (one mother did not provide information on her age) average age was 34.85 years (SD = 3.55 years), fathers’ (one father did not provide information on his age) average age was 37.10 years (SD = 4.69 years), and child’s average age was 36.51 months (SD = 1.70 months). 58.1% of the couples were married. Most participants were Swiss (79.8% of fathers, 78.2% of mothers) and German (12.1% of fathers, 16.1% of mothers). Other nationalities included Austria, Hungary, Turkey, United States, Canada, Italy, England, France, Sweden, and the Netherlands (<2.0% each). Participants were well educated with 75.8% of mothers and 66.1% of fathers having earned a university degree and the majority of mothers earned between CHF 61,000 and 80,000 per year and the majority of fathers earned between CHF 81,000 and 100,000 per year. The majority of fathers (64.5%) and mothers (45.2%) reported to have a fulltime job (100% employment). Informed consent was obtained from all subjects involved in the study.

### 2.2. Measures

#### Attachment Security and Dependency

Infant’s attachment security and dependency to mother and father was assessed by use of the Attachment Q-Sort [73]. The AQS consists of a set of 90 cards that each contain a written characteristic of child attachment behavior (e.g., “If held in parent’s arms, child stops crying and quickly recovers after being frightened or upset”) as well as affective reactions and explorative behaviors (e.g., “Child clearly shows a pattern of using parent as a base from which to explore”) in their familiar home environment. In addition, dependent behavior towards parents is included (e.g., “Child keeps track of parent’s location when he/she plays around the house”). In order to describe a particular child based on the video tapes of the conducted home visits, reliable coders sorted these 90 items equally into nine categories ranging from 1 = “not at all characteristic” through 5 = “neither characteristic nor uncharacteristic” to 9 = “very characteristic” with ten items per category depending on how characteristic these behaviors were for the observed infant. Each infant’s Q-Set was correlated with the prototypes for secure attachment and dependency [73], resulting in two scores ranging from −1.00 to 1.00 with higher scores indicating higher attachment security and higher dependency, respectively. Eight raters, who received extensive training, coded attachment security to mother and father and dependency to mother and father separately and independently. Interrater reliability was good with a mean agreement of r = 0.75 on all Q-Sort-items and a maximum mean deviation of 0.10 with regard to attachment security prototypicity.

### 2.3. Parental Report on Child Emotional and Behavioral Problems

Children’s emotional and behavioral problems were assessed by use of the Early Childhood Screening Assessment (ECSA) [74]. A German version was used for this study (SFK; “Screening Frühe Kindheit”, [75]). The SFK is a 36-item, parent report measure on young children’s emotional and behavioral symptoms (e.g., “Is irritable, easily annoyed”, “Seems nervous or worries a lot”). Child symptom items focus on internalizing and externalizing symptoms, regulatory processes, or interpersonal relationship patterns, and are rated on a Likert scale from 0 (never/rarely) to 2 (always/almost always). A total emotional and behavioral problem score is the mean score of all 36 items. The ECSA/SFK demonstrated strong convergent and criterion validity, high internal consistency, and test-retest reliability [74,76]. In the current study we used mother reports of emotional and behavioral problems (Cronbach’s α = 0.77) and father reports (Cronbach’s α = 0.80) separately.

### 2.4. Observation of Child Emotional and Behavioral Problems

Besides the parental reports on symptomatology, children’s emotional and behavioral problems were additionally assessed by objective observation using the Psychopathological Symptoms Observation (PSO) [77]. Based on the video tapes of the home visits, child symptomatology was observed during five structured and unstructured emotion eliciting situations and parent–child interactions [78]. We used the “toy removal” paradigm [79] inducing anger, the “stranger approach” and “spider” paradigms inducing fear [80], and dyadic semi-structured play interactions between each parent and the child using “Duplo” or “Play Dough” [81]. Duration of observation was about 30 min. Frequency and intensity of internalizing (anxiety and depression) and externalizing (aggression and hyperactivity) symptoms were observed using a Likert scale (0 = not observable, 1 = normative occurrence/age appropriate, 2 = sub-clinical to 3 = clinically relevant). A total emotional and behavioral problem score is the mean score of all 38 items (Cronbach’s α = 0.87).Three raters, who received extensive training, coded child symptomatology. Interrater reliability was good (Cohens Kappa > 0.65).

### 2.5. Data Analysis

Preliminary analyses and correlational analyses were performed in SPSS 28, to examine associations between attachment security, dependency, and symptoms for both parents. The Actor-Partner-Interdependence-Model (APIM) was carried out in AMOS 28, to consider the dependent structure of data and to apply a family systems perspective.

## 3. Results

### 3.1. Preliminary Analysis

Table 1 shows means, standard deviations, and correlations of all study variables. Attachment security to mother and father was significantly positively related, as was dependency to mother and father. Attachment security and dependency to one parent was also positively related. There were no significant associations between attachment security to one parent and dependency to the other parent. Maternal and paternal reports on child symptomatology were significantly positively related, whereas the observation of symptoms was only related to maternal reports on emotional and behavioral problems, but not to paternal reports (see Table 1), but the associations of mother–observer and father–observer ratings were not significantly different (z = 1.23, *p* = 0.220).

### 3.2. Main Analysis

First, we were interested in the associations between child–mother and child–father attachment security and dependency and reported as well as observed child symptoms of emotional and behavioral problems. Table 1 also shows these correlation coefficients. Attachment security to mother was significantly negatively associated with maternal reports on emotional and behavioral problems of the child and observed symptoms. Furthermore, attachment security to mother was marginally negatively related to paternal report on symptomatology. Thus, children with lower attachment security to mother showed more emotional and behavioral problems in maternal and paternal reports, as well as in the observation. Dependency to mother was marginally positively associated with maternal report and observation of child symptomatology. Thus, mothers reported more symptoms and coders rated higher symptomatology for children with higher dependency scores towards mothers. Attachment security to father was significantly negatively related to observed symptomatology, indicating, that a more secure child–father attachment goes along with less observed symptoms. Furthermore, child–father dependency was significantly positively related to maternal reports of emotional and behavioral problems of the child. Thus, mothers described children with higher dependency scores towards the father as having more emotional and behavioral problems. No other associations were found.

**Figure 2:**
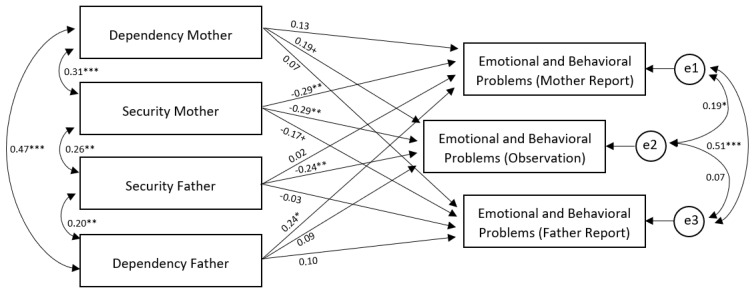
Actor and Partner Effects of Child-Parent Attachment on Parental Reports and Objective Observation of Emotional and Behavioral Problems. Note: *** *p* < 0.001, ** *p* < 0.01, * *p* < 0.05, + *p* < 0.10.

Second, we were interested in actor and partner effects of child-parent attachment on parental reports and objective observation of child emotional and behavioral problems. Figure 2 shows the APIM model for attachment security, dependency, and symptomatology. Model fit was good: χ^2^ = 1.28, df = 2; *p* = 0.527; CMIN = 1.282, *p* = 0.641; GFI = 0.997; NFI = 0.992; CFI = 1.000; RMSEA = 0.000 (90% CI = 0.000–0.157). 

The APIM shows a significant negative actor effect of child-mother attachment security on maternal reports of emotional and behavioral problems (β = −0.29, *p* = 0.002). Moreover, it shows a marginal negative partner effect of child-mother attachment security on paternal reports of emotional and behavioral problems (β = −0.17, *p* = 0.081), indicating that mother–child attachment security was negatively related with both mothers’ and fathers’ reports on emotional and behavioral problems. Furthermore, the APIM shows a significant negative partner effect of child–father dependency on maternal reports of emotional and behavioral problems (β = 0.24, *p* = 0.016). Thus, mothers report more child symptomatology when children were more dependent on their fathers. No other actor or partner effects in the APIM were significant.

With regard to observed symptomatology, the APIM shows significant negative effects of attachment security to mother (β = −0.29, *p* = 0.002) and father (β = −0.24, *p* = 0.007) on observed symptomatology. Thus, attachment security to both parents is related to less observed symptoms. Furthermore, the APIM shows a marginal positive effect of mother–child attachment dependency on observed symptoms (β = −0.19, *p* = 0.061). Thus, more symptoms were observed in children with a higher dependency to their mother.

## 4. Discussion

The main goals of this study were to examine whether child–mother and child–father attachment security and dependency and reported as well as observed child symptoms are related and whether specific actor and partner effects of attachment security and dependency to mother and father reveal differential influences on maternal and paternal ratings of symptomatology.

For many decades, only few studies examined early attachment to both mothers and fathers in the same family and its effects on child adjustment [82,83,84]. This study follows this tradition also including father’s perspective on child adjustment and considering attachment security as a relationship characteristic as well as dependency.

First, we found that attachment security was significantly positively associated with dependency in the mother–child relationship, but only marginally positively related in the father–child relationship. Thus, attachment security and dependency seem to be slightly more independent in child–father than in child-mother relationships. This would somehow support the idea that attachment to mother is characterized by a higher frequency of closer emotional interactions, whereas child–father attachment is characterized by more challenging interactions and more autonomy support [85,86]. However, this also may characterize this sample, where attachment security to mother also is characterized by increased closeness. From a theoretical perspective, attachment security and dependency are independent constructs [38]. Empirical evidence supports this theoretical assumption, revealing that the prototypic criterion sorts of dependency and attachment security represent orthogonal dimensions referring to 12-month-olds and negatively related referring to 36-month-olds [87], and show no significant associations in many empirical studies using the AQS dimensions in child-mother dyads e.g., [60,88]. Using the Strange Situation Procedure (SSP), infants classified as securely attached to mother showed less dependency as toddlers than those who were insecurely attached as infants [89,90], suggesting that secure attachment is a sign of effective self-regulation of the needs for attachment and autonomy.

The results also revealed that AQS attachment security to mother and father is moderately positively correlated similar as reported in other studies [86,91]. Moreover, AQS dependency to mother and father showed an even higher positive association, suggesting that AQS dependency might be a characteristic of the child and no relationship specific characteristic [38]. The positive correlations between the child’s AQS dimensions for mother and father are not as high to suggest that children’s attachment security and dependency to mother and father are identical. However, the results make clear that both attachment dimensions are also not completely independent. Therefore, we applied an actor–partner-interdependence model, when examining the effects on child symptoms.

Furthermore, the results of our study showed that maternal and paternal ratings of child symptomatology are significantly positively associated. This is in line with other studies in preschool [65]. Moreover, maternal ratings of emotional and behavioral problems of their children were significantly positively related to observer ratings, whereas paternal ratings were not, but the associations of mother–observer and father–observer ratings were not significantly different. Thus, in case of high scores in the observation of child symptomatology both parents describe the child as more maladjusted as well (although the mother to a higher extent). Nevertheless, all three perspectives on child symptoms are important and not identical, and reveal specific associations with attachment security and dependency, which differ for child–mother and child–father attachment relationships.

From a family systems perspective as well as from a statistical point of view, the assessed variables within families are dependent. Taking into account this statistical (and theoretical) dependence of data, we applied an actor-partner interdependence approach. 

In our study, the APIM shows actor effects of child-mother attachment security on maternal perception of child symptoms, but no actor effect of child–father attachment security on father’s perception of child symptomatology. This is in line with meta-analytic evidence, that attachment security to mother is a promotive factor for child mental health. In contrast, we found no promotive effect of attachment security to father on child mental health in father’s perception of his child. In a superficial view, this contradicts with current meta-analytic evidence on the promotive effect of child–father attachment on child outcomes [37]. However, most studies reported in the meta-analyses use maternal ratings or combined ratings of both parents on child symptoms, confounding attachment relationships and rater effect. Therefore, most of these studies show a lack of fathers’ perspectives, and in APIM thinking, report only the “partner effect“ of child–father attachment on maternal perception of child symptoms. In this study, we differentiate actor and partner effects, showing that attachment security to mother marginally effected father ratings of child problems. However, attachment security to father was not significantly associated with maternal perception of child symptomatology. Thus, the family systems perspective on attachment and child psychopathology, integrating different dyadic attachment relationships (child-mother sub-system (E) and child–father sub-system (D), see Figure 1) and attachment dimensions (attachment security and dependency) and different perceptions of child maladjustment (perspective of mother (C), father (B), and observer (H), see Figure 1) reveal other results as the individual or dyadic perspectives as reported in other studies summarized in the meta-analyses. Summarizing, our results on parental perception of child problems replicate the promotive effect of child-mother attachment security on child mental health in both paternal and maternal perception of child symptoms, but not for child–father attachment. This is different for observed symptomatology, as discussed later.

Earlier research on AQS dependency showed associations with children’s sleep problems in infancy [40,41] and more insecure ambivalent attachment classification [42]. In preschool children, high AQS dependency might also be seen as a sign of low autonomy and low effective self-regulation. Thus, dependency should be associated with more symptoms perceived by the parents. However, the APIM in our study shows no actor effects of child dependency to mother or to father on maternal or paternal ratings of child problems. Interestingly, a significant partner effect of child–father dependency on maternal ratings of child symptoms appeared. This means that mother’s individual appraisal of child’s behavior as problematic is influenced by the amount of dependency within the child–father relationship. The more dependency the child shows to the father (e.g., clinging to father, demanding behavior towards father, fussy towards father) the more problematic the mother perceived her child. This is not the case for father’s perception of his child. We conclude that mothers’ ratings of children’s problems are not only influenced by the children’s attachment security to her or her individual characteristics (e.g., maternal depression), but also by the child’s closeness and clinginess to the father. However, we did not find this effect for fathers’ ratings. Thus, if the child stays rather close to the mother seems not to be an indicator of mental health problems (e.g., anxiety) in the father’s appraisal of the child. This difference may be the result of parent-specific interaction styles and contexts for mother–child and father–child dyads. If fathers are more often (sensitively) challenging in their interaction with the child and mothers more often sensitive [92,93], then the clinging behavior to mother is more often observed compared to clinging to father and may contribute to differences in the appraisal of the child by each parent. We therefore suggest that the debate on rater discrepancies found in the assessment of child symptoms [68] should additionally consider the effect of the child’s relationship characteristics to the other parent. This may especially be the case for mother ratings, which are mainly used in research.

We additionally included the observation of children’s symptomatology by trained raters to add a more objective rating of children’s mental health problems in standardized challenging situations beside parent ratings. The results show that in a family systems perspective on attachment and psychopathology, attachment relationships to mother and father are both relevant for child mental health. Child–father as well as child-mother attachment security have a comparable promotive effect on children’s observed symptomatology supporting the theoretical assumption that attachment security to mother and father contribute to child adjustment separately. Interestingly, the relationship quality of AQS attachment security to mother and father seems to have a more important role for child adjustment compared to the more individual characteristic of the child of AQS dependency.

Results when using objective observation of child mental health problems support the attachment hypotheses that attachment security to both parents is associated with less problem behavior. Thus, the experience of external effective emotion regulation within the attachment relationship leads to less problem behavior, e.g., regulation problems. However, when relying on parental subjective perception of child mental health problems, as usually done in research, results are more sophisticated. Attachment security to mother, i.e., finding security in comforting and exploration with her conveys to both parents that the child is well regulated. In contrast, attachment security to father seems not to contribute to the parental perception of a well-regulated child. Neither for maternal nor paternal perception of child’s symptoms, attachment security in the child–father relationship (sub-system D, Figure 1) seems to be that relevant, but it is salient for the objective observation. However, the dyadic attachment security in the child-mother relationship (sub-system E, Figure 1) seems to be relevant for maternal and paternal perception as well as for the objective observed child symptoms. Thus, other child–father relationship characteristics (e.g., paternal challenging behavior, teaching behavior, exploration supportive behavior, activation-relationship) might lead to a perceived well-regulated child by parents. These father–child relationship characteristics should be included in future studies.

The results of our study may enrich existing evidence on the association between attachment and psychopathology by additionally using observation of child symptoms instead of relying on mainly maternal ratings only. Observation of child symptomatology has been successfully used in studies on the diagnostics of children’s mental health problems or their developmental precursors in early childhood [68,94,95,96,97,98]. We suggest that more attachment studies should use observational approaches not only for attachment assessment but also for the assessment of child psychopathology to control for confounding effects of parental ratings as has been shown in this and in other studies.

Although the current study yielded some important and unique findings, it also has limitations. First, participants of this study were nonclinical, mainly Caucasian two-parent families who were highly educated and had a moderate to high socioeconomic status, which limits generalization of our findings. Future research may use a clinical sample to increase variance in child symptoms and child–parent relationships. Moreover, the effects found for children’s dependency to fathers on maternal perception of child mental health problems may only be valid for Western countries. The observed closeness and clinginess to the father may have a different meaning in countries with a more collectivistic orientation compared to more individualistic countries. In collectivistic countries, children’s closeness and proximity to their caregivers is more accepted and supported, whereas autonomy support is more pronounced in countries with a more individualistic orientation [99] and also shows different outcomes in children’s inhibition and adjustment [100]. Attachment research in Asian countries such as Korea, China or Japan shows a similar proportion of secure attachment patterns as in many Western countries, but a higher proportion of insecure-ambivalent than insecure-avoidant attachment patterns in early childhood [99,101] which might be expressed in higher scores of AQS dependency. Umemura et al. [102] describe that mother’s striving for closeness to one’s baby as expressed in “amae” or “dew” is more typical for collectivistic countries. Indeed, a study with Japanese children revealed that children’s “amae” behaviors to mothers are positively associated with AQS dependency to mothers but not to attachment security [103]. Japanese and Korean mothers’ parenting is also characterized by a fast attempt to calm the child and less attempts to support the child’s independence. Moreover, some studies also show that both Chinese and Canadian mothers support more autonomy compared to connectedness in their children [104]. Thus, we suggest replications of this study in more collectivistic countries, but especially research on the effects of dependency to fathers on the perception of child mental health problems.

Second, we used concurrent data for the analyses. Future studies should use a longitudinal design with more assessment waves and a longer period for the prediction of child symptoms. Additionally, cross-lagged panel analyses may help to detect the direction of associations between attachment and child symptoms during preschool as the cross-lagged effects may not be evident during all phases of childhood. From a family systems perspective, our current analyses did not include influences of the parental sub-system (see Figure 1, sub-system F) on child adjustment (individual child sub-system A) or child-mother (sub-system E) or child–father attachment relationship (sub-system D). Influences of the parental sub-system, e.g., dyadic coping of parents [57,105], parental conflicts [106,107,108], gate keeping [109], or co-parenting [110] play an important role for child wellbeing and are often the focus of family interventions [111]. Thus, future studies should include this sub-system as well. Developmental psychopathology postulates the promotive and protective effect of attachment security for later development [9,25]. Our study supports the promotive effect of attachment security due to our non-risk sample. However, this allows no final conclusions about the protective effect of attachment security. Future studies need to address this developmental psychopathological idea using a risk sample.

## Figures and Tables

**Table 1:** Means, standard deviations, and correlations of all variables.

	M	SD	1	2	3	4	5	6
**Attachment (AQS):**								
1. Security Mother	0.61	0.18						
2. Dependency Mother	−0.01	0.20	0.28 **					
3. Security Father	0.56	0.20	0.23 *	−0.10				
4. Dependency Father	−0.06	0.19	−0.01	0.46 ***	0.16 +			
**Emotional and Behavioral Problems:**								
5. Mother Report (SFK)	0.39	0.19	−0.25 **	0.16 +	−0.03	0.30 ***		
6. Father Report (SFK)	0.43	0.19	−0.16 +	0.07	−0.06	0.13	0.54 ***	
7. Observation (PSO)	0.11	0.05	−0.29 **	0.17 +	−0.31 ***	0.14	0.29 **	0.14

Note. AQS = Attachment Q-Sort, SFK = Screening Frühe Kindheit, PSO = Psychopathological Symptoms Observation, M = Mean, SD = Standard Deviation; N = 124; *** *p* < 0.001, ** *p* < 0.01, * *p* < 0.05, + *p* < 0.10.

## Data Availability

The data presented in this study are available on reasonable request from the corresponding author.
